# Evaluating the Geographical Accessibility and Equity of COVID-19 Vaccination Sites in England

**DOI:** 10.3390/vaccines10010050

**Published:** 2021-12-30

**Authors:** Catherine Duffy, Andy Newing, Joanna Górska

**Affiliations:** 1 School of Mathematics, University of Leeds, Leeds LS2 9JT, UK; catherine.duffy@geolytix.co.uk; 2 School of Geography, University of Leeds, Leeds LS2 9JT, UK; 3 HERE Technologies, 5611 ZT Eindhoven, The Netherlands; joanna.gorska@here.com

**Keywords:** COVID-19 vaccination programme, geographical coverage, vaccination site accessibility, NHS, HERE Technologies

## Abstract

We assess the geographical accessibility of COVID-19 vaccination sites—including mass vaccination centers and community-level provision—in England utilizing open data from NHS England and detailed routing data from HERE Technologies. We aim to uncover inequity in vaccination site accessibility, highlighting small-area inequality hidden by coverage figures released by the NHS. Vaccination site accessibility measures are constructed at a neighborhood level using indicators of journey time by private and public transport. We identify inequity in vaccination-site accessibility at the neighborhood level, driven by region of residence, mode of transport (specifically availability of private transport), rural-urban geography and the availability of GP-led services. We find little evidence that accessibility to COVID-19 vaccination sites is related to underlying area-based deprivation. We highlight the importance of GP-led provision in maintaining access to vaccination services at a local level and reflect on this in the context of phase 3 of the COVID-19 vaccination programme (booster jabs) and other mass vaccination programmes.

## 1. Introduction

The UK became the first country to approve a COVID-19 vaccine, kick starting a nationwide programme of immunisation beginning on 8th December 2020 [1]. By end-September 2021, approximately 41 million people had received at least a first dose COVID-19 vaccination in England, with uptake standing at 89.7% among the population aged 16 and over [2]. The National Health Service (NHS) in England administered vaccinations (free at the point of delivery) using a phased vaccination strategy, dividing the population into cohorts based on age, vulnerability and risk as the primary drivers of vaccine roll out. Highest priority cohorts were offered vaccinations in the period December 2020 to April 2021 (phase one, including all adults over the age of 49), with phase two (the remaining adult population) commencing in April 2021 [3]. This necessitated a network of over 2000 vaccination sites and required considerable service reorganisation within the NHS at a rapid and unprecedented scale. This paper considers one core aspect of the spatial organisation of the COVID-19 vaccination programme in England, assessing the provision of vaccination sites, capturing their geographical accessibility (the ease with which potential recipients can physically access those sites). Specifically, it aims to uncover inequity in vaccination site accessibility at the neighborhood level. Analysis are based on the network of vaccination sites at the peak of phase 2 of the vaccination programme in July 2021, with recommendations made for the administration of ‘booster jabs’ (phase 3) and other routine or mass vaccination programmes. 

Health service providers such as the NHS continually make strategic decisions regarding the balance between service efficiency, the allocation of scarce resources and accessibility of facilities. With finite resources available, it is impossible to provide ubiquitous coverage of vaccination sites or any other health service which is delivered from fixed locations or which benefits from economies of scale. In a primary health care setting, facility accessibility has been defined as ‘‘the ease with which the residents in a given area can reach a particular service or facility” [4] (p. 70). Whilst there is a lack of consensus on the optimum means by which to measure geographical accessibility to any form of service provided in discrete locations (see Section 2), it is recognized that metrics capturing geographical accessibility at the small-area level reveal spatial inequalities that can be addressed by policy makers and service providers.

In seeking to vaccinate large population sub-groups—some of which are elderly, extremely vulnerable or suffer limited mobility or financial resources-in a very short period, the geographical accessibility of vaccination sites is important in the efficient administration of COVID-19 vaccinations. Depending on the vaccine used, most recipients initially required two doses administered 8 to 12 weeks apart, with recipients invited to book their vaccination appointments via an online and telephone ‘National Booking Service’ (NBS), enabling them to choose a preferred vaccination site and appointment dates/times based on local availability. Some patients were also contacted directly by their registered General Practitioner (GP) with the option to receive their vaccination at a local GP surgery (which may not be their regular surgery) if their surgery was part of a primary care network-led vaccination service. Under this scheme, vaccines were administered at a single GP surgery on behalf of a network of local GPs, hereafter termed ‘GP-led’. Vaccinations were also administered at hospitals (predominantly targeting in-patients and staff), residential care settings, pharmacies, community medical facilities and also via mass vaccination centers specifically set up for this programme, some capable of vaccinating thousands of people each day. In this paper, a ‘vaccination site’ refers to any location administering COVID-19 vaccines, whilst a ‘vaccination center’ is a specific category of vaccination site offering vaccinations at scale—typically thousands per day. 

Whilst NHS England claimed that 99% of the population lived within 10 miles of a vaccination site at the peak of the vaccination programme in July 2021 [5], the media reported challenges that recipients faced in attending vaccination appointments. These included lack of available appointments in many localities, e.g., see [6] or difficulty reaching sites due to lack of transport [7]. Notions of coverage (as reported by NHS England [5]) suggest a level of equity in the provision of vaccination sites. However, the lack of justification for a 10-mile threshold and the failure to consider accessibility—the ease with which those sites can be reached—could hide considerable geographical inequity in provision, which are considered throughout this manuscript.

Whilst there are many studies of vaccination uptake for COVID-19 (including articles within this Special Issue) and for other routine vaccination programmes, there are a deficit of studies on vaccination site accessibility. In an isolated international example, Guhlincozzi and Lotfata [8] consider accessibility of COVID-19 and flu vaccination centers in Chicago for elderly and disabled population sub-groups, classing individuals as having access if they lived within a mile of their nearest vaccination site. Other research on the topic of COVID-19 vaccination centers has been mostly speculative, such as the evaluation of multiple hypothetical scenarios of vaccination site networks in New Zealand [9]. It is expected that as vaccination programmes increase and more data become available, the volume of published literature will increase. 

Even routine forms of vaccination—such as the annual winter flu programme, or vaccinations predominantly offered to travelers—are notably absent from studies of health service accessibility, with the only known example related to accessibility of Yellow Fever vaccination sites [10]. Studies specifically related to vaccination site accessibility may be limited because those vaccines are typically administered via existing health facilities such as GP surgeries, for which a series of accessibility metrics do exist [11]. In relation to those vaccination programmes, the COVID-19 vaccination programme is unique in that its population coverage (ultimately seeking near complete uptake by all adults) and temporal scale (rapid roll out over a period of months) required development of a comprehensive network of additional sites, which were not entirely drawn from the existing stock of primary or preventative healthcare services. 

The following sections draw on the unique context of the COVID-19 vaccination rollout in England to assess—at the neighborhood level—the accessibility of COVID-19 vaccination sites. Section 2 outlines approaches that can be used to measure accessibility, with a detailed overview of the demand and supply side data used in this study. Section 3 presents an overview of our findings, considering notions of equity in vaccination-site accessibility as driven by location (including region of residence, rural-urban geography and availability of GP-led services). Section 4 and Section 5 discuss our findings and their implications for future vaccination programmes including phase 3 of the COVID-19 vaccination programme (booster jabs), recognising that improvements in the accessibility of vaccine sites could enhance immunisation uptake, coverage and ultimately the success of mass vaccination programmes. 

## 2. Materials and Methods

Applied research across a variety of domains and international contexts have given rise to a number of approaches to capture geographical accessibility, which can be calculated at the level of individual facilities (‘how accessible is this vaccination site?’) or small geographic areas (‘how accessible are vaccination sites to the households in this neighborhood?’). Approaches fit into two broad categories- ‘distance-based’ and ‘container-based’ [4]. Distance-based methods consider the ‘cost’ of making a journey (e.g., between an individuals’ home and their nearest vaccination site) using straight-line distance or-most commonly travel time. There are a number of applications of distance-based approaches in studies of health service provision at a variety of spatial scales in the UK [12,13,14] and in an international context [15,16,17]. 

By contrast, container-based methods calculate supply to demand ratios within a geographic ‘container’ (often administrative or statistical boundaries for which demographic information are readily available). In spite of the ease with which these metrics can be interpreted and compared (between areas or over time), container approaches have faced criticism due to their lack of consideration of distance and the inability of these methods to simulate flows (e.g., of patients) outside of the administrative or statistical zone within which they reside [4]. Whilst methods do exist which combine distance- and container-based approaches; in particular, floating catchment area (FCA) and kernel density (KD) models (see Yang et al.) [18], their data requirements for model building and calibration extend beyond the data publically available for this research (specifically vaccination site-level information on capacity). Driven by data availability and the specific aims of this study—to uncover neighborhood level inequity in vaccination site accessibility utilizing measures that could be calculated on a national scale-distance-based methods are employed as outlined in the following sub-sections. 

The analysis undertaken in this paper considers only England owing to differences in both data availability and vaccination strategy between devolved nations of the UK. Data capturing the supply side (vaccination site locations) were obtained from NHS England. Demand side data were drawn from the 2011 Census of Population and Housing in England and Wales alongside corresponding geographic data for neighborhoods, considered in turn in the following sub-sections. 

### 2.1. Supply Side—Vaccination Site Provision 

Vaccination site data were supplied by NHS England. They were first published on 11 January 2021, with NHS England claiming that they would be updated weekly. Whilst weekly updates were not actually published, the data were regularly amended through to 26 July 2021 and accessed via a Freedom of Information request. With each update, NHS England revised their ‘coverage’ figure-the proportion of adults living within ten miles (16.1 km) of a COVID-19 vaccination site. Although the format of the vaccination site data did change slightly over time, each dataset included the name, address and postcode of each vaccination site. The data released on 22 January 2021, and each subsequent iteration also split the vaccination sites into categories capturing the site type, including hospital hubs, GP-led vaccination services, pharmacies and vaccination centers. In Section 3, we very briefly present a summary of these data to illustrate the growth in the vaccination site network and reported coverage. 

Most of the analysis presented in this paper uses the list of vaccination sites published on 6 July 2021. At that time—during the peak of phase 2 of the vaccination programme-2091 vaccination sites were listed [5]. Five records related to planned vaccination centers that had not yet opened or had no recorded postcode and these were removed from the data. Duplicate records were removed, most commonly occurring due to decommissioned sites and their replacements appearing in the same dataset, or GP surgeries inside medical centers being reported as two separate sites. There were nine cases of vaccination sites recorded as both a vaccination center and GP-led service and these were retained only as vaccination centers. Geographic co-ordinates were appended to each vaccination site (based on its postcode) using postcode centroids derived from the ONS Postcode Directory. 

In England, as each age or risk-based cohort became eligible for the vaccine they were invited to book an appointment via the online ‘National Booking Service’ (NBS) or via a direct invitation from their registered GP. Although not reported fully here due to space constraints, the list of vaccination sites within the NHS Data was interrogated against the list of available vaccination sites presented to an individual when booking their vaccination. This revealed that hospital hubs should not be included as they gave priority for vaccinating hospital staff and in-patients and were generally not available for public appointments. All 274 hospital hubs were removed from the analysis. As a result, analysis was undertaken using 191 vaccination centers, 691 pharmacies and 911 GP-led vaccination sites within the supply side dataset. It also revealed that many (but not all) GP-led services were only available to patients registered at that practice. The analysis was also repeated without inclusion of the 911 GP-led services (as many of these are only available to registered patients), highlighting the impact of the eligibility of GP-led services on overall vaccination site accessibility. 

### 2.2. Demand Side 

Households (which contain populations eligible for COVID-19 vaccination) are nested within geographical zones (which we term neighborhoods) which can be attached to a series of neighborhood indicators of population size and composition, car ownership, degree of rurality and measures of area-deprivation. We use Lower Layer Super Output Areas (LSOAs) to represent neighborhoods. LSOAs are an area-based statistical and administrative geography designed to be as consistent as possible in terms of population size and composition, containing between 400 and 1200 households [19]. 

**Figure 1:**
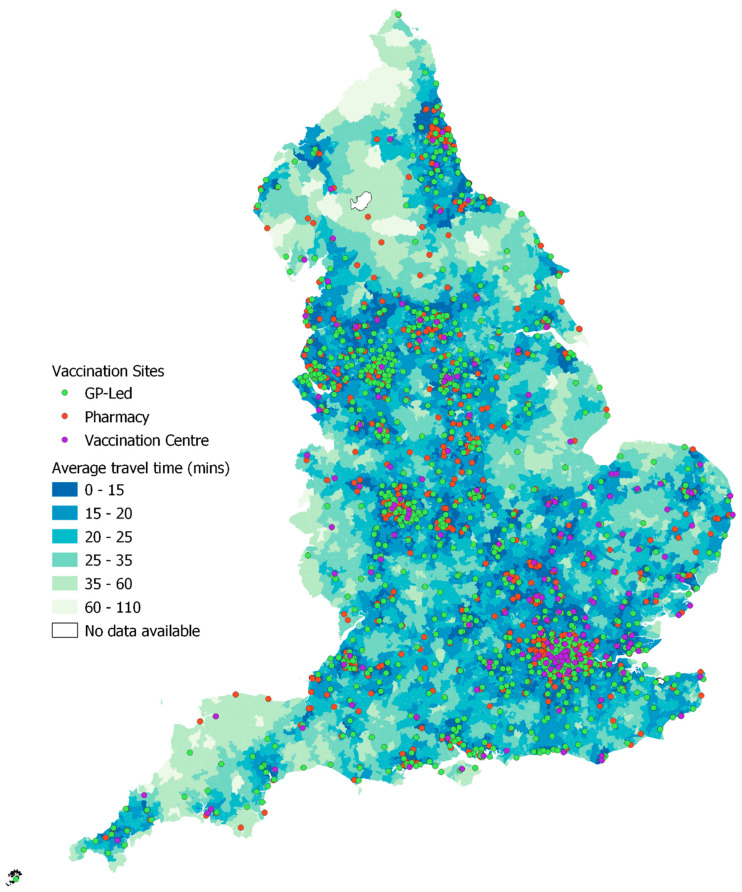
Average journey times to closest five vaccination sites by all modes of transport (weighted to account for inferred usage based on LSOA level split between car and public transport).

Whilst LSOAs represent geographic zones (and are depicted as such in Figure 1), analysis utilized LSOA population-weighted centroids (PWCs) [20]-a single point representing that LSOA-from which distances or journey times to facilities (in this case COVID-19 vaccination sites) can be calculated. PWCs account for the distribution of population within that LSOA and give a more accurate representation of journey time (than alternative geometric centroids) for households within that LSOA. This is especially important for rural LSOAs which may stretch across a large geographic extent, yet the population within it may be clustered in a hamlet or along a single stretch of road. 

We attached additional attribute information to each LSOA for use in subsequent analysis. This included the total adult population (aged 18+), drawn from the 2011 Census of Population and Housing in England and Wales. At the time of analysis, these were the age groups able to book a vaccination appointment via the NBS. Attribute information capturing an area-based classification of urbanity or rurality—the Office for National Statistics Rural Urban Classification for Small Area Geographies [21] were appended to each LSOA to understand how accessibility varies across urban and rural geographies. The 2019 English Indices of Deprivation (IoD) [22] were also appended to each LSOA, enabling us to use this area-based measure of relative deprivation to consider the relationship between neighborhood disadvantage and vaccination site accessibility. It should be noted, however, that the IoD do themselves contain a measure of access to local services including GP surgeries. For some analysis we also consider how our modelled journey times vary by region, utilizing the nine English regions (formerly known as Government Offices for the Regions), which act as the highest tier of sub-national division in England and are widely used for comparative statistical analysis. 

The journey time data used for these analysis (see Section 2.3) enable routes to be calculated by mode of transport (car, public transport or on foot). To accurately represent the most likely mode of transport individuals would use to attend a vaccination site, a transport mode was assigned to every member of the population. This was applied based on the proportion of households who self-reported having use of a car at the time of the 2011 Census on an LSOA by LSOA basis [23], Table QS416EW. These car ownership rates were applied to the 2019 adult population in each LSOA, generating an estimate of the number of vaccine-eligible individuals who would most likely travel by private car, as either a driver or passenger, and a count of individuals inferred to access vaccination sites (and other services) on foot or using public transport. It is acknowledged that a proportion of these individuals would actually access these services using other means, which could include lifts from friends or relatives or bespoke transportation organized by the NHS and other local organizations. Nevertheless, this step ensures that our accessibility calculations are not overly skewed by comparatively poor accessibility in predominantly remote areas where public transport is non-existent but high car ownership rates mean that access barriers may be limited. 

### 2.3. Linking Supply and Demand-Capturing Vaccination Site Accessibility 

Once supply and demand side data had been prepared, the bulk of the analysis was based on modelled journey times between neighborhoods (using PWCs) and vaccination sites. The analysis presented here considers the most accessible five vaccination sites to each LSOA. This recognizes that many people will not have received their vaccination at their closest site due to appointment availability, opening hours, personal convenience and a range of other individual and locally specific factors. In particular, many individuals may have been willing to travel further than their most accessible site in order to attend a mass vaccination center (generally offering a greater number of appointments and extended opening hours). This also reflects that many GP-led services were running on different timescales to the NBS (with many individuals receiving their NBS invitation prior to an invitation from their GP-led service), meaning that many individuals would not have accessed their vaccination at their most accessible site if that was a GP-led service. 

Travel times and travel distances were calculated for the interaction between each LSOA PWC and its most accessible five vaccination sites using HERE Technologies’ Routing and Public Transit APIs (both version 8). HERE is an established spatial intelligence provider who provide location data, including those data used within this analysis, under license to a number of major end-users which include Garmin, Facebook and Amazon [24]. By combining extensive travel time matrices for various transport modes with traffic volume data, public transport stop locations and timetables, their APIs calculate complex routes and return travel time and journey segment information at a high level of accuracy [24]. The Public Transit API uses public transit agency data, external services and data collected by HERE to discover public transit options, request public transit routes, and transit-related information. To extract data from the API, string functions were used to generate a URL for each record (representing a PWC-vaccination site pair) to be passed through the API. The API also enables a departure time to be set in order to reflect typical traffic conditions. A 17:30 p.m. departure on a Tuesday (avoiding school holidays) was set in order to reflect a level of congestion associated with one of the peak travel periods during a typical working day and therefore ensure that journey times were realistic. 

Records (origin-destination pairs) for which no journey time was returned were checked manually. For car transport these affected only 10 routes (from a total of 329,062 requests) and were attributed to a single island for which travel time to the mainland could not be calculated. A greater number of routes (3877) could not be calculated within the Public Transit API (which makes use of all available public transport options within a given locality—e.g., public bus and train services). In many cases this was because the vaccination site was too close to the origin for public transport to be a viable option, and in these cases walking time (taken from the pedestrian transport mode in the HERE Routing API) was used instead. Where routes were multi-modal (e.g., pedestrian segments between home and bus stop; interchange between transport services) these were summed to report a single total journey time value. 

The following section presents outputs from the journey time analysis, linking neighborhood-level vaccination site accessibility to the underlying demand side data highlighted in Section 2.2., including rurality and deprivation. It also considers the impact on accessibility if GP-led services are excluded from the analysis, to simulate the more limited availability of these services, as discussed fully in Section 4. 

## 3. Results

The number of sites providing COVID-19 vaccinations increased considerably during the phase 1 and phase 2 vaccination programme to July 2021 (Table 1), with the network (and its NHS-reported coverage of >99% of households within 10 miles) largely established by mid-April. The marginal increase in coverage between the initial network (January 2021) and the established network (July 2021) suggests that development of additional sites during that period focused predominantly on adding capacity rather than improving accessibility. The high reported coverage suggests that accessibility has not changed considerably during the vaccination programme and so an analysis of vaccination site accessibility at different time points has not been undertaken. As noted in Section 2, analysis subsequently presented here is based on the established network as reported by the NHS on 6 July 2021 [5]. 

NHS analysis summarized in Table 1 highlights their claims that 99.11% of the adult population in England lived within a straight-line distance of 10 miles of a vaccination site on 6 July 2021. Our analysis, using sophisticated routing data, suggests that 98.5% of eligible adults lived within a ‘drive distance’ of 10 miles from their nearest vaccination site, supporting the NHS’ headline coverage figures. However, determining accessibility using coverage as a proxy (proportion of the population within a given threshold distance) has considerable shortcomings and fails to justify the 10-mile threshold or consider the ease with which those sites can be reached, potentially hiding considerable geographical inequity in accessibility. The following sub-sections thus present more sophisticated analysis of vaccination site accessibility (as of July 2021) accounting for journey times (rather than distance), mode of transport and capturing a more realistic measure of provision. As outlined fully in Section 2, this approach captures more realistic measures of physical site accessibility, recognizing the importance of choice of vaccination site to account for lack of appointment availability, eligibility (e.g., patients registered with a given GP practice only) and opening hours, all of which may be barriers to using the closest or most accessible sites. 

### 3.1. Journey Time Analysis at the LSOA Level

Figure 1 illustrates overall vaccination site accessibility at the neighborhood level for all LSOAs in England as of 6 July 2021. It shows calculated median journey time for households resident in each LSOA and utilizes both private (car) and public (walking, bus, train) transport. As outlined fully in Section 2, residents of each LSOA that are eligible for the vaccine (adult population) have been allocated to each mode of transport (public vs. private transport) based on their inferred transport availability, using self-reported car ownership rates. Average journey time to vaccination centers varies quite considerably across England, with Figure 1 highlighting comparatively lower journey times in major cities such as London, Birmingham, Manchester, Leeds and Newcastle. Areas characterised by high average travel times are evident in North Devon (South West) and along the borders with Wales and Scotland (See Appendix A for a reference map). There are also many LSOAs with high travel times in the northern counties of Cumbria, Northumberland and County Durham, coinciding with several remote rural areas including the North Pennines and North York Moors. Additionally, longer travel times are experienced by some households in rural Lincolnshire, Cambridgeshire and Norfolk, along with more isolated pockets of poorer accessibility on the south coast. Figure 1 suggests that areas with poorer accessibility tend to be those that are more rural and remote from the major population centers. It also suggests that vaccination sites show a tendency to be clustered around major urban areas, which may drive additional inequalities in provision and accessibility between urban and rural areas, as explored in Section 3.2. 

Travel times for each neighborhood were calculated as the mean journey time from the LSOA population-weighted centroid to its closest five sites, to reflect that residents may not have booked into their closest site due to personal choice, lack of appointments or ineligibility (the limitations of GP-led services were discussed above). For those households with access to private transport, the median value of these travel times is just over 10 min. This suggests that the average drive time between any neighborhood (LSOA) in England and its five most accessible vaccination sites is approximately 10 min. The 75th percentile is just 13 min and 45 s and the maximum average journey time for all LSOAs is just under 58 min. All households with access to private transport therefore have at least one vaccination site that can be reached within less than an hours’ drive, whilst most have considerable choice of sites within this threshold, representing excellent access to vaccination sites.

**Figure 2:**
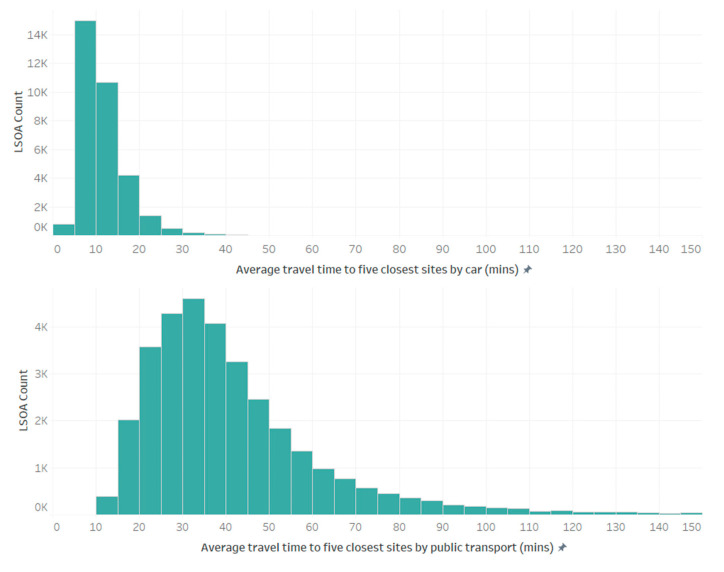
Histograms comparing average journey times to closest five vaccination sites by private car and public transport on an LSOA by LSOA basis.

As summarised in Section 1, households without access to private transport faced additional challenges in accessing vaccination sites, with analysis presented by Burn-Murdoch and Neville [7] suggesting that at the start of the vaccination programme (January 2021), approx. 360,000 eligible adults without a car lived over an hours’ journey (on foot or by public transport) to their nearest vaccination site. Our analysis, utilizing HERE’s high quality public transport routing data, highlights that over 97% of the population do live within a one-hour journey (incorporating time spent walking to service stops/stations and waiting time) of their nearest vaccination site by public transport. However, Figure 2 illustrates the stark difference in average travel time to the closest 5 vaccination sites by LSOA, broken down by mode of transport. Public transport users experience a median travel time that is 25 min longer than those with access to private car transport. Although not illustrated here, analysis reveals geographical variations in accessibility to vaccination sites by public transport, with higher travel times experienced in many rural areas, considered further in Section 3.2 alongside regional variations in vaccination site accessibility. 

### 3.2. Regional and Rural-Urban Inequalities in Vaccination Site Accessibility

**Figure 3:**
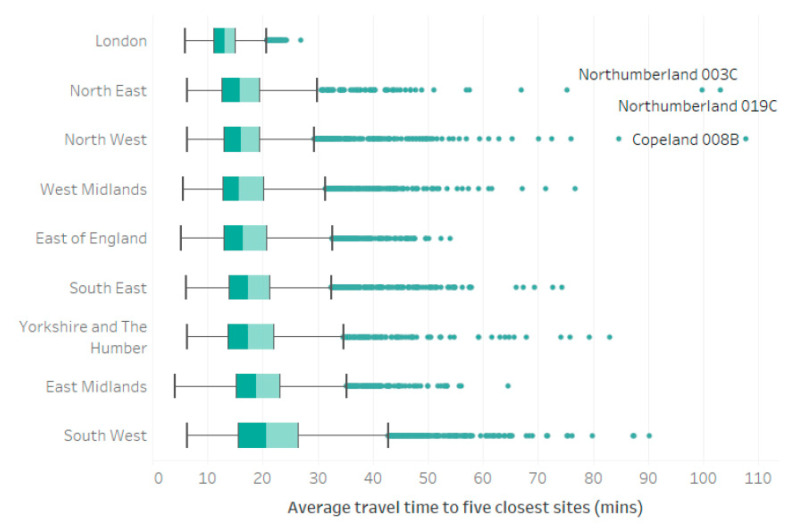
Average journey times to five closest vaccination sites by all modes of transport (weighted to account for inferred usage based on LSOA level split between car and public transport), grouped by region.

Whilst private transport affords ‘better’ access to vaccination sites (quicker journey to nearest site and greater choice of sites available within threshold distances), there remain inequalities between neighborhoods which hold true irrespective of the mode of transport used. These can be illustrated at a variety of spatial scales and are considered here by region. Figure 3 highlights that residents of London enjoy the best access to vaccination sites (based on travel time), alongside the least intra-region variability in accessibility, with a narrow spread and few outliers. Whilst residents of the North East and North West regions also exhibit some of the lowest average journey times to vaccination sites, they also contain some of the more extreme outliers. This is characteristic of the geography of those regions which contain some of England’s largest cities (e.g., Manchester and Newcastle) alongside remote rural areas (Northumberland and Cumbria), with associated challenges in providing accessible services in both densely populated urban areas and sprawling rural areas with low-population densities and a legacy of comparatively poor public transport provision. The South West exhibits the ‘poorest’ access, with median travel times greater than any other region and a ‘whisker’ which is approximately 1.5 times the IQR, largely driven by journeys which extend to an average of 90 min for residents of some LSOAs with heavy reliance on public transport and highlighting considerable intra-regional variation in vaccination site accessibility. 

**Figure 4:**
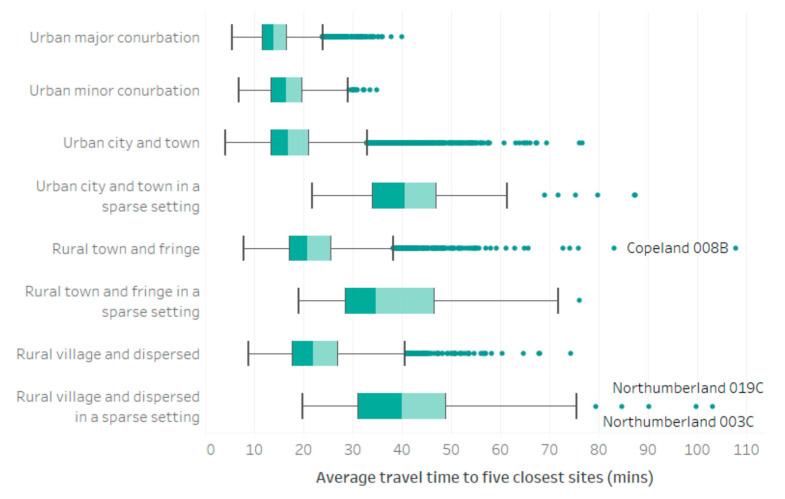
Average journey times to five closest vaccination sites (accounting for both private and public transport, weighted according to inferred usage), grouped by rural-urban classification at the LSOA level.

Inter and intra regional inequalities revealed by Figure 3 suggest that degree of urbanity influence accessibility to vaccination sites, with some of the longest journey times recorded in those regions traditionally associated with large remote rural populations. Drawing on the rural-urban classification of LSOAs [21], Figure 4 illustrates average journey times to vaccination sites by rural-urban classification, weighted according to the inferred split of private and public transport usage. Our analysis reveals that raw travel times to vaccination sites increase as rurality increases, with median journey time (to the closest 5 sites) by public transport just 14 min in the largest urban centers, increasing to 40 min in the most remote ‘rural village and dispersed in a sparse setting’ neighborhoods. This is in-line with expectations given the lower density populations, longer journey times to service centers and more limited or nonexistent public transport provision associated with many rural areas, though it should be noted that higher rates of car ownership are evidenced in the most remote LSOAs (compared to many urban neighborhoods) and are accounted for within our analysis. Additionally, there is a clear impact of population density on vaccination site accessibility, with the longest journey times exhibited by residents of neighborhoods classified as being ‘in a sparse setting’, irrespective of whether they fall within a predominantly urban or rural area. 

### 3.3. Impact of GP-Led Provision and Deprivation on Vaccination Site Accessibility

Section 3.2 highlights the link between population density and vaccination site accessibility, with neighborhoods in a ‘sparse’ or ‘dispersed’ setting typically experiencing poorer accessibility in the form of greater average journey times and heightened inequality between neighborhoods. Population density inevitably influences both the proximity of local services and the availability of public transport, both of which are predominantly concentrated in larger population centers, which thus serve as local service centers. Figure 1 reveals that whilst residents of major urban areas benefit from access to a range of community-based vaccination sites (those hosted by GP surgeries and pharmacies for example), they also have access to mass-vaccination sites, some of which have capacity to provide in excess of 2000 vaccinations per day [25]. As noted in Section 2.1, GP-led services were typically only available to patients registered with that GP, with invitations for GP-led appointments often received after their NBS invitation [26], meaning that many individuals received their vaccine via a non-GP-led service. 

To account for some of these vagaries around GP-led provision we undertook analysis, which simulated the removal of these services, in order to illustrate the important role that GP sites play in the provision of an accessible vaccination service. Our analysis reveals that once GP-led services are removed, the average adult faces a journey time that increases by 7 min, with increases of up to 15 min in some of the most remote rural areas. Removal of GP-led services reveals 2463 LSOAs from which the closest site was over an hours’ journey by public transport. Census data reveal that—within those neighborhoods—a total of almost 380,000 adults live in a household without access to a car. This echoes concerns raised at the beginning of the vaccination programme [7]. Less than 40% of the adult population have five or more vaccination sites accessible within an hour by public transport when GP-led services are excluded. In comparison to private transport, the median travel time to the most assessable 5 vaccination sites by public transport is 35 min higher when GP-led services are excluded. Removal of GP-led services thus suggests that inequalities in vaccination site accessibility are driven by mode of transport (specifically availability of private transport) and exacerbated by eligibility for GP-led services (typically only bookable by patients registered with that GP).

Availability of private transport, degree of rurality and access to GP practices are also indicators used (among many others) to capture area-based measures of deprivation. Whilst literature and wider policy (including established measures of area deprivation [22]) recognize the link between deprivation and accessibility of services—including healthcare (see Page et al. [27] for an overview), we find no evidence for inequalities in COVID-19 vaccination site accessibility driven by underlying neighborhood-level deprivation. Thus, whilst our analysis reveals a series of important drivers of inequalities in vaccination site accessibility, there is no evidence that these are driven or exacerbated by area-level deprivation. In light of these findings, Section 4 considers the importance of GP-led services, transport availability and rural-urban Geography in the assessment of vaccination site accessibility.

## 4. Discussion

The COVID-19 vaccination programme in England aimed to vaccinate as many of the eligible population as possible in a short period of time, bringing notions of accessibility and coverage to the fore on the premise that increasing the number of vaccination sites should help improve geographic accessibility and reduce access-driven inequalities. As highlighted by our analysis, the average drive time between any neighborhood (LSOA) in England and its five most accessible vaccination sites is approximately 10 min and the maximum average journey time for all LSOAs is just under 58 min. This suggests that overall, geographical accessibility of the vaccination site network is excellent and compares very favourably with other forms of primary, secondary and emergency care see for example [13,27,28]. 

In spite of NHS-published coverage figures which implied that almost all households lived within 10 miles of a vaccination site, our findings support media coverage, e.g., see [7] reporting challenges in accessing vaccination sites, especially among some less mobile or disadvantaged groups, including those reliant on public transport. Irrespective of the availability of GP-led services—discussed further below—we uncover considerable access inequalities driven by the availability of public transport. This is in keeping with broader headline findings in relation to local service accessibility reported by the National Audit Office (NAO) [28] who specifically identify the additional access burdens faced in reaching GP services (for routine appointments rather than vaccinations) by households reliant on public transport. In our analysis, many households reliant on public transport faced lengthy journeys—in some cases prohibitively so—with 842 LSOAs, containing a total population of 92,408 households who lack access to private transport, facing journey times in excess of 1 h. This especially true within parts of rural South West England (Devon and Cornwall), Northern England (notably Northumberland, Cumbria and North Yorkshire), Lincolnshire and the Welsh border. 

Capturing travel time to the five most accessible sites allows vaccine recipients to exercise some personal choice over which vaccination site they visit (e.g., to take account of available appointment times) and highlights that lack of appointment availability at the most accessible site did not typically preclude accessibility. However, removal of GP-led services—which were typically available only to patients registered at that specific practice—does increase journey times, especially for those recipients who are reliant on public transport. This highlights that GP-led services are a very important component of the vaccination site network, especially in rural localities which may already be more remote from mass vaccination centers or other forms of community provision (e.g., pharmacies). During Phase 2 of the vaccination programme, there was considerable ambiguity around the availability of vaccinations from some GP-led sites, in part due to a lack of coordination between GP-led services and the NBS. This resulted in duplicated invites for many patients, with the faster paced roll out of invites via the NBS resulting in many patients travelling further than required to obtain vaccines via mass vaccination centers when they could have waited for an invitation for a vaccination appointment at their usual GP surgery [26]. 

Whilst mass vaccination centres may address concerns regarding capacity (ability to vaccinate thousands per day), their centralized locations do not necessarily promote equity in accessibility. In spite of the points raised above, Haynes [29] reported that in the early stages of the vaccination programme, over three quarters of vaccinations were administered at GP-led services, suggesting patient-preference to receive their vaccine at a local site. However, as surgeries were still expected to cope with ‘business-as-usual’ appointments on top of COVID-19 vaccinations, some GPs reported being overworked, to the extent that some PCNs opted out of involvement in Phase 2 of the vaccination programme [30], potentially worsening accessibility among proximate neighborhoods. 

Although not directly related to accessibility, one of the key recommendations from this study is for a comprehensive and all-encompassing national booking system for future mass-vaccination programmes so that recipients have the opportunity to book an appointment at their most accessible sites, irrespective of the mode of delivery. Given the important role of GP surgeries in providing accessible vaccination sites in many localities, it is inevitable that the challenges and delays in being able to book vaccinations at these sites (even where eligible, i.e., to registered patients) resulted in vaccine recipients travelling further than required to receive their vaccination(s). In recommendations to the charity sector, the International Longevity Centre (ILC) suggest that vaccination uptake among some under-represented groups could be addressed via improvements to vaccination-site accessibility, yet their only practical recommendations for doing so involve better use of taxis or lift-sharing services and better signposting of vaccination opportunities [31]. This paper argues that whilst these approaches may incrementally improve awareness of and access to existing vaccination sites, improvements to vaccination accessibility is best achieved by minimizing access inequalities via effective design of the network of vaccination sites, making best available use of existing accessible health care settings, such as GP surgeries.

In September 2021, phase 3 of the vaccination programme began in England, enabling individuals who had received their second dose COVID-19 vaccination at least 6 months beforehand to obtain a booster dose, again administered via GP-led services alongside vaccination centers and other community provision, including pharmacies. As with phase 1 and 2 of the vaccination programme, invites are sent by GPs alongside the NBS, and availability is also provided via a series of walk-in sites (for which no appointment is required). Whilst this range of provision may provide a number of opportunities for an eligible individual to obtain their booster vaccination, it may again result in individuals travelling further than required if there is a time lag in sending out invitations for local GP-led services. In common with phases 1 and 2 of the vaccination programme, GPs were not invited to administer COVID-19 booster jabs at the GP practice level, with clusters of GP practices (within primary care networks) favored given the economies of scale that can be achieved at these larger sites. The NHS cited considerable operational and logistical challenges associated with vaccination administration at a GP practice level (summarized in [32]). O’Dowd [33] reports considerable frustrations on the part of GPs who wish to be able to administer the COVID-19 booster jab at their own surgeries as part of routine appointments with patients or in conjunction with the GP and pharmacy led annual winter flu vaccination campaign. Given the important role that GP-led services played in maximizing accessibility to vaccination sites during phase 2 of the vaccination programme (as highlighted in our findings), it is inevitable that the decision not to enable booster jab delivery from all GP surgeries misses an opportunity to further improve accessibility to vaccination sites. This is especially true in rural or sparsely populated areas who are most remote from the centralized mass vaccination centers or primary care network GP-led services. 

## 5. Conclusions 

COVID-19 has highlighted the importance of effective vaccination programmes in promoting public health. This study, undertaken at the peak of phase 2 of the COVID-19 vaccination programme in England (July 2021), evaluates geographical accessibility of COVID-19 vaccination sites and highlights the importance of private transport and area type (broadly urban/rural) in driving inequalities in vaccine site accessibility at a neighborhood level. We highlight the important role GP surgeries play in maintaining ‘good’ access to proximate vaccination sites, especially in remote rural localities and for those most-reliant on public transport. Whilst overall accessibility of vaccination sites is considered excellent, challenges in accessing these services among some population subgroups should not be overlooked in ongoing and future vaccination campaigns. 

We note that the decision not to offer phase 3 of the vaccination programme at an individual GP practice level misses a tremendous opportunity to improve access to these services. We also highlight the benefits of utilizing appropriate measures to capture key accessibility metrics and note deficiencies in NHS England’s [5] use of crude coverage measures. We recommend moving away from simple measures of coverage (x households within y distance of nearest site) in favor of more complex (yet computationally straightforward) measures which account for journey time, mode of transport and recipient choice of vaccination site (to account for opening hours, appointment availability or eligibility criteria at certain sites, such as GPs). 

Timely analysis of these nature is only possible due to the availability of publicly available data on vaccination site provision, alongside user-friendly and robust tools through which accessibility (in this case journey time) can be measured. Arribas-Bel et al. [34] highlighted—at a range of spatial scales ranging from local applications to international comparisons—the importance of high quality, timely and open data related to the COVID-19 response, enabling evidence based-insight. Should more data become available regarding the vaccination programme in England—including vaccination site capacity and appointment availability, or information capturing actual demand-supply interactions (which vaccination sites were used by individuals)—then there is considerable scope to further extend these analyses and enhance the ongoing provision of COVID booster vaccinations. 

## Figures and Tables

**Table 1:** Number of vaccination site locations (excluding hospital hubs) and NHS-reported coverage (percentage of the adult population within ten miles (16.1 km) of a vaccination site). Drawn from selected iterations of the NHS England published COVID-19 vaccination site lists between January and July 2021.

Date Published by NHS England	Number of Sites	Coverage (% of Households)
10 January 2021	785	96.00
12 February 2021	1330	97.26
12 March 2021	1409	Unreported
23 April 2021	1528	99.07
28 May 2021	1715	99.11
6 July 2021	1814	99.11

**Figure A1:**
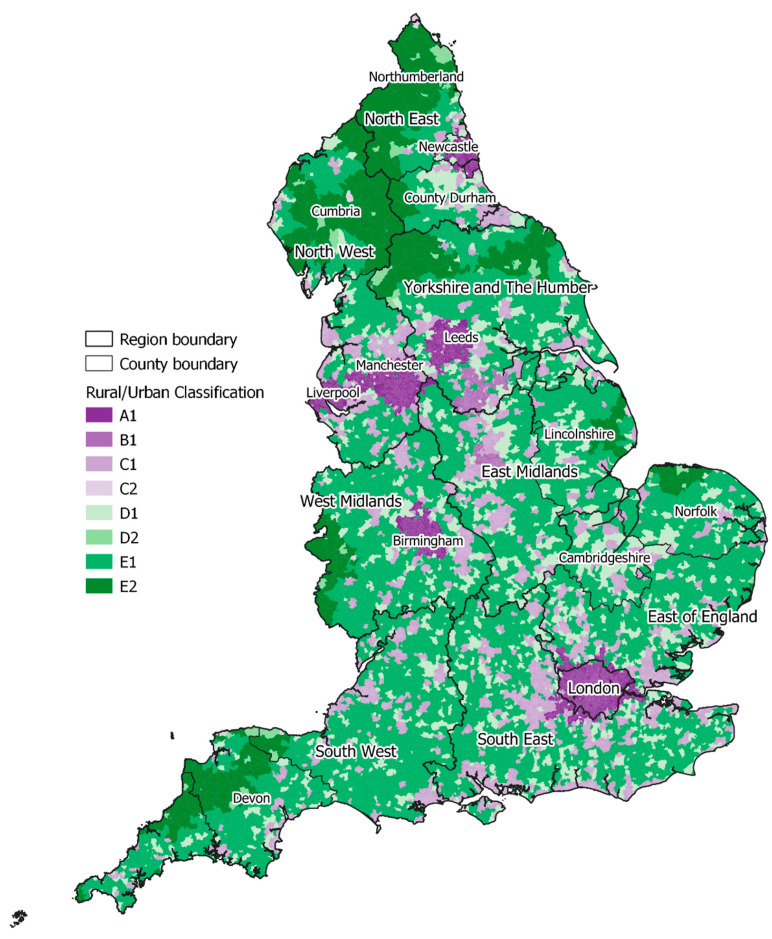
Reference and contextual map to support the discussion in Section 3 and Section 4. Rural/Urban classification [21] captures degree of rurality or urbanity using 8 categories: A1-Major Conurbation; B2-Minor Conurbation; C1-City and Town; C2-City and Town in a sparse setting; D1-Town and Fringe; D2-Town and Fringe in a sparse setting; E1-Village; E2-Village in a sparse setting.

## Data Availability

Demand and supply side data used in this study are publically available, as highlighted in Section 2. Analysis was undertaken using APIs supplied by HERE Technologies. These are commercial products available to other users via license from HERE Technologies.

## Appendix A

Figure A1 serves as a reference map, highlighting regions, counties and named localities discussed in Section 3 and Section 4, alongside the rural-urban classification for small area geographies [21].
